# Inappropriate implantable cardioverter defibrillator shocks—incidence, effect, and implications for driver licensing

**DOI:** 10.1007/s10840-017-0272-4

**Published:** 2017-07-20

**Authors:** Eiichi Watanabe, Katsunori Okajima, Akira Shimane, Tomoya Ozawa, Tetsuyuki Manaka, Itsuro Morishima, Toru Asai, Masahiko Takagi, Toshihiro Honda, Atsunobu Kasai, Eitaro Fujii, Kohei Yamashiro, Ritsuko Kohno, Haruhiko Abe, Takashi Noda, Takashi Kurita, Shigeyuki Watanabe, Hiroya Ohmori, Takashi Nitta, Yoshifusa Aizawa, Ken Kiyono, Ken Okumura

**Affiliations:** 1 Department of Cardiology, Fujita Health University School of Medicine, Dengakugakubo 1-98, Kutsukake-cho, Toyoake, Aichi 470-1192 Japan; 2 Department of Cardiology, Himeji Cardiovascular Center, Himeji, Japan; 3 Department of Cardiovascular and Respiratory Medicine, Shiga University of Medical Science, Otsu, Japan; 4 Department of Cardiology, Tokyo Women’s Medical University, Tokyo, Japan; 5 Department of Cardiology, Ogaki Municipal Hospital, Ogaki, Japan; 6 Department of Cardiology, Ichinomiya Municipal Hospital, Ichinomiya, Japan; 7 Department of Cardiovascular Medicine, Osaka City University Graduate School of Medicine, Osaka, Japan; 8 Division of Cardiology, Saiseikai Kumamoto Hospital Cardiovascular Center, Kumamoto, Japan; 9 Department of Cardiology, Ise Red Cross Hospital, Ise, Japan; 10 Department of Cardiology and Nephrology, Mie University Graduate School of Medicine, Tsu, Japan; 11 Cardiovascular Medicine, Toyohashi Heart Center, Toyohashi, Japan; 12 Department of Heart Rhythm Management, University of Occupational and Environmental Health, Kitakyushu, Japan; 13 Department of Cardiovascular Medicine, National Cerebral and Cardiovascular Center, Suita, Japan; 14 Department of Medicine, Faculty of Medicine, Division of Cardiovascular Center, Kinki University School of Medicine, Osaka-Sayama, Osaka, Japan; 15 Department of Cardiology, Tsukuba University Hospital Mito Education and Training Center, Mito, Japan; 16 Department of Cardiovascular Surgery, Nippon Medical School, Tokyo, Japan; 17 Department of Research and Development, Tachikawa Medical Center, Nagaoka, Japan; 18 Division of Bioengineering, Graduate School of Engineering Science, Osaka University, Toyonaka, Japan; 19 Department of Cardiology, Hirosaki University Graduate School of Medicine, Hirosaki, Japan

**Keywords:** Arrhythmia, Syncope, Driving, Implantable cardioverter defibrillator, Prevention

## Abstract

**Purpose:**

Patients with implantable cardioverter defibrillators (ICDs) have an ongoing risk of sudden incapacitation that may cause traffic accidents. However, there are limited data on the magnitude of this risk after inappropriate ICD therapies. We studied the rate of syncope associated with inappropriate ICD therapies to provide a scientific basis for formulating driving restrictions.

**Methods:**

Inappropriate ICD therapy event data between 1997 and 2014 from 50 Japanese institutions were analyzed retrospectively. The annual risk of harm (RH) to others posed by a driver with an ICD was calculated for private driving habits. We used a commonly employed annual RH to others of 5 in 100,000 (0.005%) as an acceptable risk threshold.

**Results:**

Of the 4089 patients, 772 inappropriate ICD therapies occurred in 417 patients (age 61 ± 15 years, 74% male, and 65% secondary prevention). Patients experiencing inappropriate therapies had a mean number of 1.8 ± 1.5 therapy episodes during a median follow-up period of 3.9 years. No significant differences were found in the age, sex, or number of inappropriate therapies between patients receiving ICDs for primary or secondary prevention. Only three patients (0.7%) experienced syncope associated with inappropriate therapies. The maximum annual RH to others after the first therapy in primary and secondary prevention patients was calculated to be 0.11 in 100,000 and 0.12 in 100,000, respectively.

**Conclusions:**

We found that the annual RH from driving was far below the commonly cited acceptable risk threshold. Our data provide useful information to supplement current recommendations on driving restrictions in ICD patients with private driving habits.

**Electronic supplementary material:**

The online version of this article (doi:10.1007/s10840-017-0272-4) contains supplementary material, which is available to authorized users.

## Introduction

Implantable cardioverter defibrillators (ICDs) improve survival in patients at risk of sudden cardiac death [1]. However, these patients have an ongoing risk of sudden incapacitation that may cause harm to themselves and others when driving [2–10]. An obvious concern is the effect of arrhythmias and/or discharges of devices on a patient’s level of consciousness and ability to drive. According to the literature, the rate of syncope or loss of consciousness associated with appropriate ICD therapy varies from 2 to 15% [5–7, 11–13]. Approximately 10–20% of ICD patients experience inappropriate ICD therapies (therapies delivered for non-ventricular arrhythmias) according to the previous reports [11, 14–16]; however, few studies have studied the rate of syncope or loss of consciousness as a result of inappropriate therapy. Thus, a large variation exists among countries concerning driving restrictions following inappropriate ICD therapies [17–22].

Currently, inappropriate therapy reduction programming, including higher detection rates, longer detection intervals, and optimized supraventricular tachycardia discriminators, have been reported to reduce ICD shocks without increasing the arrhythmic syncope among ICD patients for primary prevention [12, 23–26]. Although such shock reduction programming is beneficial, it may take time for this programming to spread to the actual clinical setting.

It is critical to collect data regarding the rate of the incidence that lead to impaired driving after inappropriate ICD therapy to prevent serious driving accidents, while at the same time avoiding unnecessary driving restrictions on patients with ICDs for both primary prevention and secondary prevention. We set two objectives: (1) to identify the rate and causes of syncopal episodes associated with inappropriate ICD therapy and (2) to calculate the risk of harm to other road users in order to provide a scientific basis for driving restrictions in ICD patients.

## Methods

We analyzed the inappropriate ICD therapy event data between 1997 and 2014. This retrospective, observational, non-randomized study was conducted at 50 institutions participating in prospective ICD studies [27, 28] and Japanese Heart Rhythm Society board-certified institutions (participants’ list in Supplementary File). The ethics committee of each institution approved the study protocol, and all patients gave their written informed consent. The study complied with the Declaration of Helsinki and its later amendments or comparable ethical standards.

### Database and ICD therapy event analysis

The Japanese Heart Rhythm Society board-certified electrophysiologists at the 50 institutions were asked to submit a total number of ICD implantation and case report form for the patients who had inappropriate therapies after the initial implantation. It included the patient demographics, underlying disease, comorbidities, ventricular function, and medications. The data regarding the ICD included the date and indication for the implantation, date and time of the onset of any appropriate or inappropriate therapies, cause, and type of inappropriate therapy, in addition to the activity associated with the inappropriate therapy. Finally, we asked them whether or not each inappropriate ICD therapy was associated with syncope. The implanted system manufacturers were Biotronik (Berlin, Germany), Boston Scientific (St. Paul, MN, USA), Medtronic (Minneapolis, MN, USA), and St. Jude Medical (St. Paul, MN, USA). All ICDs in this study were equipped with intracardiac electrogram (EGM) storage. The ICDs were interrogated every 3 months and when clinically appropriate, such as after a delivered therapy. ICD therapies were defined as either antitachycardia pacing (ATP) or shock therapy, including cardioversion and defibrillation. Therapies were categorized as appropriate when they occurred in response to ventricular tachycardia (VT) or ventricular fibrillation (VF) and as inappropriate. An inappropriate therapy included therapy for atrial fibrillation (AF), supraventricular tachycardias (SVTs), including sinus tachycardia, abnormal sensing (T wave oversensing, myopotential, and electromagnetic interference, other than oversensing of short VV intervals related to lead fractures), and lead failures [11, 14]. The determination of an inappropriate or appropriate ICD therapy was made by the electrophysiologists at the participating institutions and was not adjudicated by independent electrophysiologists. The ICD programming was left to the discretion of each investigator. We did not exclude patients in whom the generator or leads were replaced. A syncopal event associated with inappropriate therapies was the primary endpoint and was defined as a total loss of consciousness with spontaneous recovery. These events were identified through reviewing the medical charts. We excluded syncopal episodes not related to inappropriate therapies. The time to the first inappropriate therapy was measured from the date of the ICD implant to the date of an inappropriate ICD therapy. The time to the second therapy was measured from the date of the first inappropriate ICD therapy to the date of the second inappropriate therapy. Regarding the second therapy analysis, only subsequent inappropriate therapies occurring >24 h after the first inappropriate therapy were considered to be second therapies.

### Risk assessment

The Canadian Cardiovascular Society Consensus Conference published a “risk of harm” (RH) formula to quantify the level of risk to drivers with ICDs according to the Ontario Road Safety Annual Report [8, 29]. It has been used in several other reports [5, 9, 16, 19, 22, 30]. The following equation is the risk of harm formula: RH = TD × V × SCI × Ac, which calculates the yearly RH to other road users posed by a driver with heart disease; TD equals the proportion of time the patient spends driving during the year (0.04 for private drivers, 0.25 for commercial drivers); V is a vehicle-specific constant based on the type of vehicle driven (1.0 for a commercial heavy truck and 0.28 for a standard-size passenger car); SCI is the annual probability of sudden incapacitation, and Ac is the probability of injury or an accident after the SCI. We used an Ac of 0.02 according to the previous studies [2, 3]. In this study, the yearly risk of SCI was calculated to be 0.13 for primary prevention and 0.14 for secondary prevention, respectively, based on the incidence of syncope associated with inappropriate ICD therapies (i.e., 1 out of 144 patients for primary prevention and 2 out of 273 patients for secondary prevention) divided by the mean follow-up period of 5.3 years. Standard errors were derived from the binomial distribution, and the 95% confidence interval was constructed with the normal approximation according to the previous study [9]. We calculated the RH after the first and second shocks, respectively, along with the previous study [9]. An acceptable RH was defined to be 5/100,000 or 0.005% [8, 9, 16].

### Statistical analysis

The *χ* ^2^ test or Fisher exact test was used for categorical data, and a Student’s *t* test or Mann–Whitney test was used for continuous variables. Comparisons of the time to therapy were made using the Kaplan–Meier method and compared with the log-rank test. Quantitative data are expressed as the mean ± standard deviation (SD) values or median with an inter-quartile range. A two-tailed *p* value of <0.05 was considered significant. Statistical analyses were performed using JMP 10.0.2 software (SAS Institute, USA) and R Project for Statistical Computing 3.2.2.

## Results

### Clinical characteristics of the patients

The baseline characteristics of the patients are summarized in Table 1. Of the 4089 patients, 772 inappropriate ICD therapies occurred in 417 patients (age 61 ± 15 years, 74% male, and the reason for the implantation was secondary prevention in 65%). No significant differences were noted in the age, sex, and baseline cardiac rhythms between the primary and secondary prevention patients. There was a significant difference in the prevalence of single-chamber or dual-chamber ICDs and cardiac resynchronization therapy with defibrillators (CRT-Ds) between the primary and secondary prevention patients. Coronary artery disease was observed in 20% of patients, and the mean left ventricular ejection fraction was 45%. The median date of the implantation was November 2010, and approximately 90% of the patients received ICDs after 2005 (supplementary figure).

**Table 1:** Clinical characteristics of the patients

Characteristic	All patients (*n* = 417)	Primary prevention (*n* = 144)	Secondary prevention (*n* = 273)	*p* value
Age (years)	61 ± 15	60 ± 15	61 ± 16	0.70
Male, *n* (%)	309 (74)	105 (73)	204 (76)	0.47
Baseline rhythm, *n* (%)				0.39
Sinus rhythm	333 (80)	118 (82)	215 (80)	
Atrial fibrillation	71 (17)	24 (17)	47 (18)	
Pacemaker escape rhythm	8 (2)	1 (0.7)	7 (3)	
Devices, *n* (%)				<0.001
Single-chamber ICD	54 (13)	17 (12)	37 (14)	
Dual-chamber ICD	284 (68)	81 (56)	203 (74)	
CRT-D	79 (19)	47 (33)	32 (12)	
Underlying heart disease, *n* (%)				
Coronary artery disease	84 (20)	19 (13)	65 (24)	<0.01
Brugada syndrome	31 (7)	16 (11)	15 (6)	0.04
Dilated cardiomyopathy	95 (23)	52 (36)	43 (16)	<0.001
Hypertrophic cardiomyopathy	52 (12)	20 (14)	32 (12)	0.57
Sarcoidosis	17 (4)	5 (3)	12 (4)	0.62
Amyloidosis	1 (0.2)	0 (0)	1 (0.3)	0.75
Long-QT syndrome	5 (1)	2 (1.4)	3 (1.1)	0.81
ARVC	15 (2)	1 (0.7)	14 (5)	0.04
VHD	9 (2)	2 (1.4)	7 (3)	0.64
Medical comorbidities, *n* (%)				
Hypertension	160 (39)	56 (39)	104 (39)	0.99
Diabetes	69 (17)	20 (14)	49 (18)	0.25
Stroke	16 (3)	11 (8)	5 (1.5)	<0.01
Chronic kidney disease	43 (10)	10 (7)	33 (32)	0.09
Left ventricular ejection fraction (%)	45 ± 17	42 ± 19	47 ± 16	<0.05
Medications, *n* (%)				
Beta-blocker	268 (64)	93 (65)	175 (65)	0.88
ACE-I/ARB	216 (52)	82 (57)	134 (50)	0.18
Amiodarone	144 (35)	36 (25)	108 (40)	<0.01
Sotalol	18 (4)	3 (2)	15 (6)	0.09
Aspirin	82 (20)	19 (13)	63 (24)	0.01
Warfarin	158 (38)	53 (37)	105 (38)	0.63

Data represent the number, frequency, or means ± SD. Chronic kidney disease = estimated glomerular filtration rate <60 mL/min/1.73 m^2^

*ICD* implantable cardioverter defibrillator, *CRT-D* cardiac resynchronization therapy with defibrillator, *ARVC* arrhythmogenic right ventricular cardiomyopathy, *VHD* valvular heart disease, *ACE-I* angiotensin-converting enzyme inhibitor, *ARB* angiotensin II type 1 receptor blocker

### Inappropriate ICD therapies

**Fig. 1:**
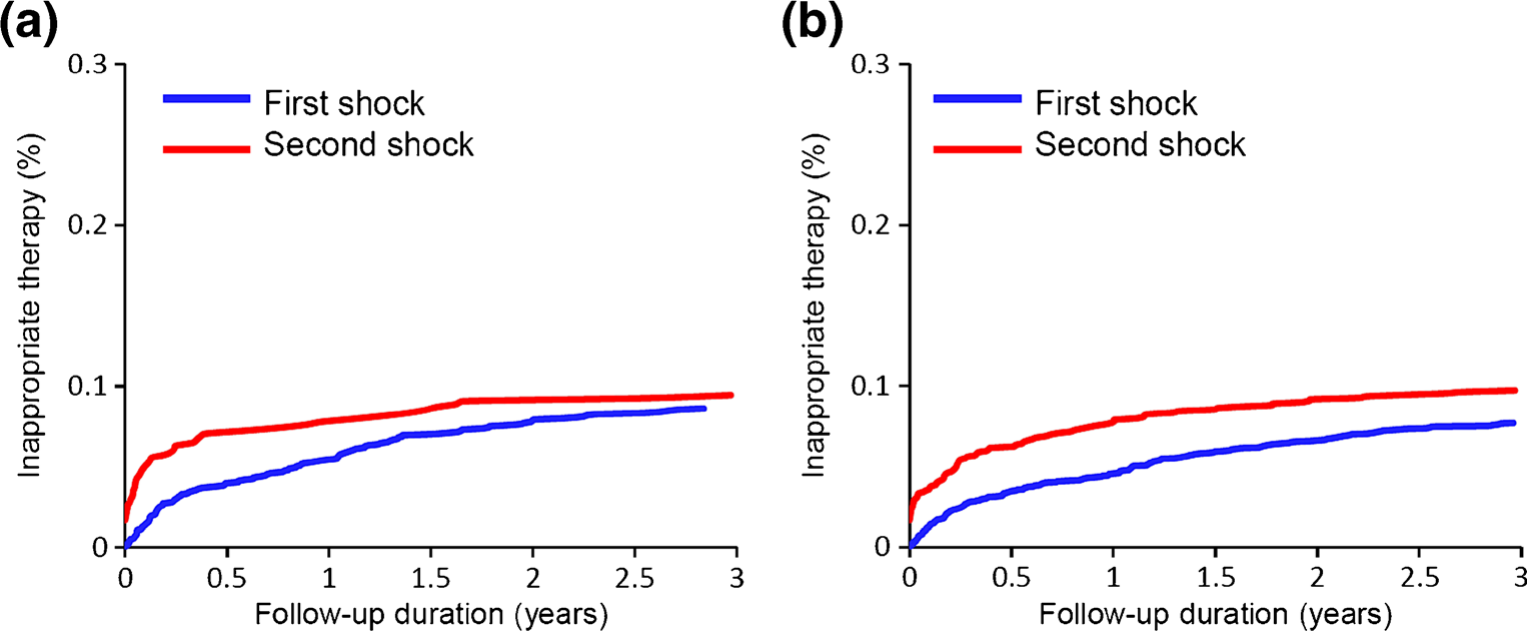
The time-dependent occurrence of an inappropriate therapy. **a** Primary prevention. The first inappropriate therapy occurred at a median time of 314 days (inter-quartile range, 64 to 697 days) after the implantation. The median time between the first and second inappropriate therapies was 85 days. **b** Secondary prevention. The first inappropriate therapy occurred at a median time of 401 days (inter-quartile range 97 to 1040 days) after the implantation. The median time between the first and second inappropriate therapies was 123 days

Patients experiencing inappropriate therapies had a mean number of 1.8 ± 1.5 inappropriate therapy episodes during a median follow-up period of 3.9 [inter-quartile range, 3.1 to 6.8] years (Table 2). A total of 169 patients (41%) had more than 1 inappropriate therapy, with a maximum of 15 inappropriate therapies. There were no significant differences in the number of inappropriate therapies between the primary prevention and secondary prevention patients (1.7 ± 1.7 vs. 1.9 ± 1.6, *p* = 0.13). The first inappropriate therapy occurred a median of 382 days after the implantation (inter-quartile range, 85 to 841 days). The median time between the first and second inappropriate therapies was 117 days (inter-quartile range, 25 to 408 days). There was a significant difference in the time to the first inappropriate therapy between the primary prevention and secondary prevention patients (median 314 vs. 401 days, *p* < 0.01). The time-dependent occurrence of the first and second inappropriate therapies for primary and secondary prevention is shown in Fig. 1.

**Table 2:** Inappropriate ICD therapy and rate of syncope

	All patients (*n* = 417)	Primary prevention (*n* = 144)	Secondary prevention (*n* = 273)	*p* value
Inappropriate therapy episodes, *n* (%)	1.8 ± 1.5	1.7 ± 1.7	1.9 ± 1.6	0.13
Number of inappropriate therapy episodes, *n* (%)				0.56
1	248 (59)	88 (61)	160 (59)	
2	102 (24)	40 (28)	62 (23)	
3	24 (6)	5 (3)	19 (7)	
4	18 (4)	5 (3)	13 (5)	
5	8 (2)	2 (1)	6 (2)	
≥6	17 (4)	5 (3)	12 (4)	
Appropriate therapy episodes, *n* (%)	150 (36)	42 (29)	108 (40)	0.03
Syncope, *n* (%)	3 (0.7)	1 (0.7)	2 (0.7)	0.96

Data represent the number, frequency, or means ± SD

The total number of inappropriate therapies by the mechanism rather than by the patient and the subclassifications of inappropriate therapies are shown in Table 3. SVT was the most common mechanism for an inappropriate therapy (63%), followed by AF (27%) and abnormal sensing (7%). A small percentage of rhythms triggering ICD therapies (2%) were unclassified. There was no significant difference in the type of inappropriate therapies between the primary and secondary prevention patients. In this study, 373 patients (89%) had one mechanism of inappropriate therapy, 38 (9%) had two mechanisms, and 6 (1%) experienced all three mechanisms.

**Table 3:** Inappropriate ICD therapy episodes

	Total therapy episodes (*n* = 772)	Primary prevention (*n* = 251)	Secondary prevention (*n* = 521)	*p* value
Cause of inappropriate therapy, *n* (%)				
SVT	483 (63)	152 (61)	331 (64)	0.42
AF	207 (27)	71 (28)	136 (26)	0.52
Abnormal sensing	52 (7)	21 (8)	31 (6)	0.21
Lead failure	15 (2)	5 (2)	10 (2)	0.95
Unclassified	15 (2)	2 (1)	13 (3)	0.11
Type of inappropriate therapy, *n* (%)				
ATP only	356 (46)	115 (46)	241 (46)	0.91
Shock only	188 (24)	64 (25)	124 (24)	0.61
ATP + shock	228 (30)	72 (29)	156 (30)	0.72
Activity associated with the inappropriate therapy, *n* (%)				0.13
Sleeping	28 (4)	10 (4)	18 (4)	
Sedentary/awake	302 (39)	112 (45)	190 (36)	
Limited exercise	59 (8)	23 (9)	36 (7)	
Moderate exercise	157 (20)	45 (18)	112 (21)	
Driving	1 (0.1)	1 (0.4)	0 (0)	
Drinking	11 (1)	3 (1)	8 (2)	
Bathing	16 (2)	3 (1)	13 (3)	
Unknown	198 (26)	54 (22)	144 (28)	
Inappropriate therapy-induced ventricular arrhythmia, *n* (%)				
VT	5 (0.6)	1 (0.4)	4 (0.8)	0.54
VF	12 (2)	4 (2)	8 (2)	0.95

Data represent the number and frequency

*SVT* supraventricular tachycardia, *AF* atrial fibrillation, *ATP* antitachycardia pacing, *VT* ventricular tachycardia, *VF* ventricular fibrillation

### Rate of syncope and risk of harm

**Fig. 2:**
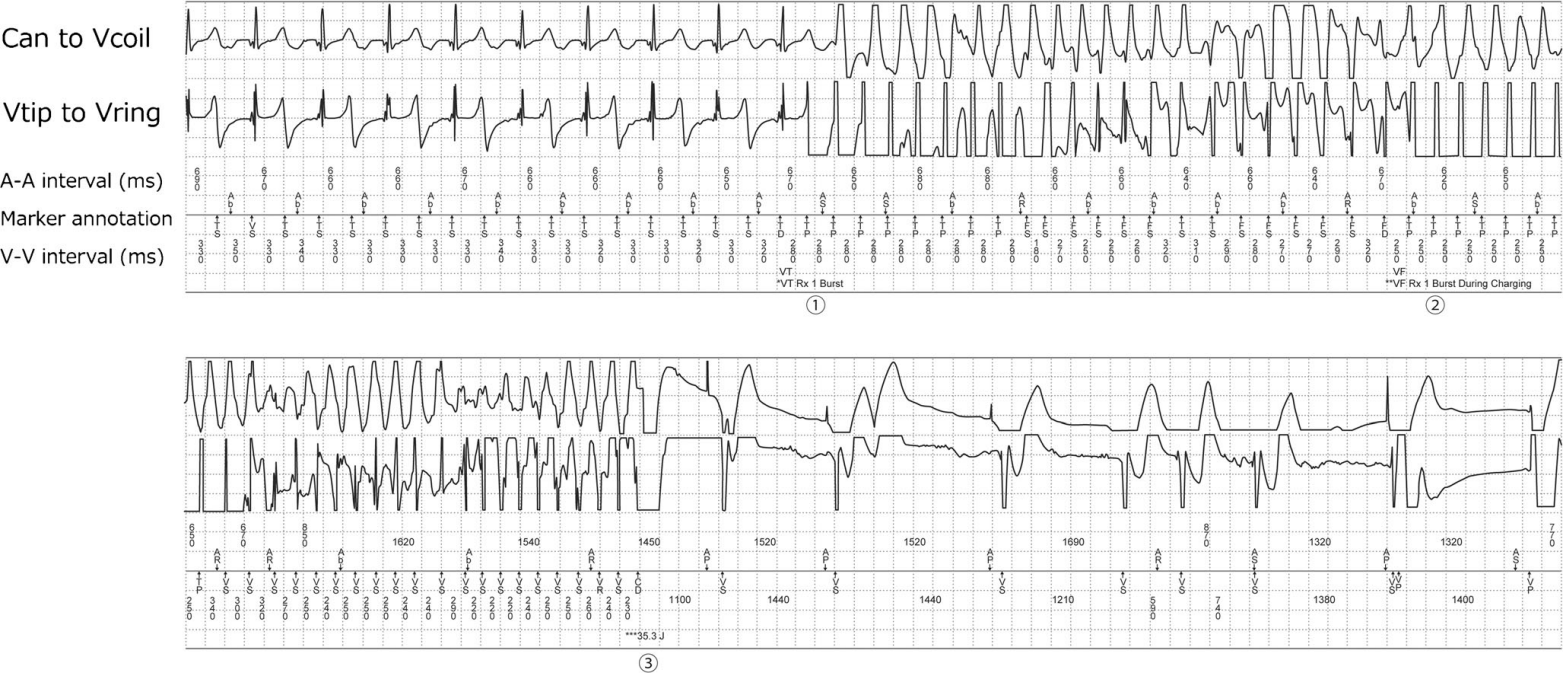
An ICD-stored intracardiac electrogram. A 61-year-old man that had dilated cardiomyopathy and chronic hemodialysis received an ICD (Secura DR, Medtronic) for secondary prevention in 2009. In 2011, he had chest discomfort and unconsciousness, followed by a shock delivery at work. A stored electrogram shows that the sinus tachycardia with T wave oversensing with a cycle length of 330 ms triggers a burst of ATP (① VT Rx 1 Burst, cycle length of 280 ms). This results in an acceleration to a tachycardia with a 250 to 320-ms cycle length, with a subsequent burst of ATP (② VF Rx 1 Burst During Charging, cycle length of 250 ms). This therapy degenerated the VT into VF, which required shock therapy for termination (③ 35.3 J). His creatinine was 12.1 mg/dL, and serum potassium was 7.0 mmol/L at admission. The upper and lower electrograms are continuous recordings. *ATP* antitachycardia pacing, *VT* ventricular tachycardia, *VF* ventricular fibrillation

**Fig. 3:**
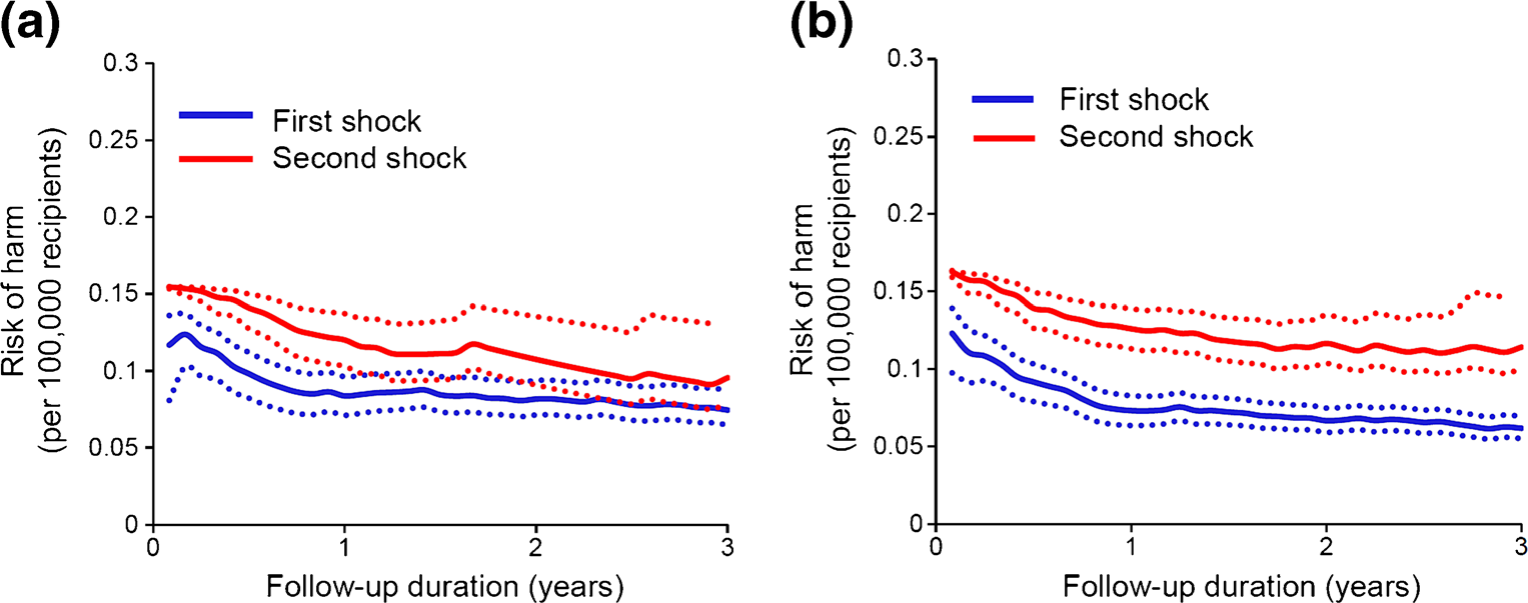
The annual risk of harm from an inappropriate ICD therapy. The risk of harm (*solid lines*) is calculated in years following the implantation or following the first inappropriate shock. The *dotted lines* represent the 95% confidence interval. **a** Primary prevention. Driving is acceptable directly following an implantation (*blue line*) (0.11/100,000) or following the first inappropriate shock (*red line*) (0.15/100,000). **b** Secondary prevention. Driving is acceptable directly following an implantation (*blue line*) (0.12/100,000) or following the first inappropriate shock (*red line*) (0.16/100,000)

Three patients (0.7%) experienced syncope associated with inappropriate therapies. Two of these patients had syncope, one during an SVT and the other during sinus rhythm and oversensing, both of which degenerated to VF, as a result of the inappropriate therapies. The latter case is presented in Fig. 2. The remaining patient who was implanted with a dual-chamber ICD for primary prevention had syncope due to AF with a rapid ventricular response, which was terminated by shock therapy. These three syncope patients did not have any further episodes of syncope with ICD therapies. In this study, only one patient experienced a shock for SVT while driving a motor vehicle, but did not experience any syncope nor cause an accident on the road. Further, no patients had any syncope or deaths related to motor vehicle accidents. For private driving habits, the maximum annual RH of the first and second inappropriate therapies in the primary prevention patients was calculated as 0.12 in 100,000 and 0.15 in 100,000, respectively. Also, that in the secondary prevention patients was calculated as 0.12 in 100,000 and 0.16 in 100,000, respectively. These RH values were found to be far below the acceptable level of 5 in 100,000 (Fig. 3).

## Discussion

In this study, we presented data on the RH posed by individuals with ICDs toward other users of the road based on the rate of syncope associated with an inappropriate ICD therapy. We showed that the annual RH from driving was far below the commonly cited acceptable risk threshold. Our data may provide useful information to supplement the current recommendations on driving restrictions in ICD patients with private driving habits.

The mean age of the study patients was 61 years, which was comparable to a previous observational study by van Ree et al. [15] (mean age, 61 years) that characterized inappropriate ICD therapies and a recent randomized study of the MADIT-RIT trial [25] (mean age of the conventional group, 63 years), but somewhat older than that in the subgroup analysis of the SCD-HeFT trial [31] (median age, 57 years). Further, in this study, the rate of single-chamber ICDs in primary prevention was 12%, which was lower than that in the recent ICD registries ranging from 23 to 39% [32, 33]. The potential explanation for the differences in the age and lower use of single-chamber ICDs for primary prevention may be found within the evolving and expanding guidelines for the implantation of ICDs over a 17-year period, device programming, and (non-) pharmacological treatment of arrhythmias or other unknown confounders. Our data indicated that the most common cause of inappropriate therapy was SVT, which included sinus tachycardia, followed by AF. We made every effort to identify the mechanism of the SVT, but missing or incomplete data hampered the complete clarification of the SVT.

There is a wide variation in the driving restrictions after inappropriate ICD therapies among countries. In the USA, scientific statements showed that a private driver must refrain from driving for 6 months after receiving any ICD therapy, regardless of whether the therapy is appropriate or inappropriate [17, 18]. In the UK, a patient must cease from driving for 1 month after the cause of inappropriate therapy has been corrected [20]. In Europe, no time frame is set; however, the patient is not allowed to drive until the cause of the inappropriate therapy is resolved [19]. According to the previous Japanese driving restrictions, ICD patients were advised not to drive for 12 months after receiving either appropriate or inappropriate therapies [21], but from 2017, ICD patients do not have to refrain from driving after receiving an inappropriate ICD therapy if it is not associated with a loss of consciousness [22].

Two recent studies calculated the RH using the data on the incidence of syncope associated with appropriate therapy [9, 30]. Merchant et al. showed that if they used a contemporary estimate for syncope associated with an appropriate ICD shock of 14%, the RH fell below the threshold at 1 month after an initial shock [30]. Few reports, however, have calculated the RH because the data on the rate of inappropriate ICD therapy-related syncope are scarce. To the best of our knowledge, for the first time, we have provided data demonstrating that the maximum annual RH posed by ICD drivers to other users of the road after a first therapy for primary and second prevention was far below the cutoff level proposed by the Canadian consensus [8]. The RH formula was developed in order to quantify the level of risk for drivers with cardiac disorders and to provide an objective and theoretical method for assigning risk to a driver [8]. The RH analysis was performed based upon the data from the early Ontario Road Safety Annual Report [29]; it is important to understand that there may be some differences among geographic areas due to the variation in the population density, driving habits, and type of vehicles. Although we have no data on the driving parameters (i.e., TD, V, or Ac) in Japan, it is feasible to implement contemporary area-specific driving parameters to calculate the RH to other users of the road. Driving restrictions are useful to protect the society from harm, but they should be balanced against the QOL of the ICD patients, which may be reduced by unnecessary driving restrictions. To accomplish this, up to date clinical evidence is required.

In this study, there were 5 episodes of VT and 12 episodes of VF as a result of inappropriate ICD therapies. Of those, two patients lost consciousness due to inappropriate ICD therapies for SVTs or abnormal sensing degenerating into VF. While ATP is highly effective in terminating VT and lowers the use of high-energy shocks, ATP may degenerate stable arrhythmias into VF and hence result in incapacity prior to the delivery of the shock [11, 34, 35]. A prospective study of 770 primary and secondary prevention ICD patients revealed that in patients receiving ATP for termination of a fast VT, syncope occurred in 0.2% of cases [35]. In the PITAGORA ICD trial, the incidence of a fast VT-related syncope following ATP was 0.97% [36]. These observations were similar to the rates observed in our study.

In this study, one patient experienced a shock while driving a motor vehicle, but did not report any syncope nor cause a traffic accident. An observational study of 241 ICD patients followed for 36 months found that 5% of secondary prevention patients had ICD shocks while driving but did not report any syncope during these shocks [6]. Data from the Antiarrhythmics Versus Implantable Defibrillators trial showed that 8% of drivers experienced ICD shocks while driving without leading to an accident on the road [11]. According to an early survey of ICD implanting physicians in the USA, there were a total of 30 motor vehicle accidents related to shocks from ICDs over a 12-year period [4]. Of those, nine were fatal accidents (eight patients with ICDs died); further, the estimated motor vehicle fatality rate for patients with ICDs of 7.5/100,000 patient-years was significantly lower than that for the general population (18.4/100,000 patient-years). From these results, we can conclude that ICD patients with secondary prevention may experience vehicle accidents due to arrhythmias or ICD discharges, but the occurrence rate is quite small, and the relative safety of driving with ICDs is supported [19, 37]. Few studies, however, have specifically examined the rate of syncope or ICD discharges while driving in patients receiving ICDs for primary prevention.

### Study limitations

This study was a retrospective observation, and there are confounders associated with such a study method. Underreporting is another limitation since the occurrence of syncope was determined by only a chart review. It has been well documented that patients often drive despite instructions not to do so, and it can be anticipated that they do not tell doctors about symptoms if they think reporting would lead to curtailment of their driving. Because of the long time span, going back to 1997, there is a heterogeneous patient population with regard to programming, but it is certainly encouraging to see a low event rate given the change in the detection algorithms and recommendations for programming for a shock reduction that has occurred in the more recent years. Data regarding the device therapies have been reported by the participating electrophysiologists and were not adjudicated by an independent committee. ICD programming was left to the discretion of each investigator. We could not assess the drivers’ licensing, driving habits, and driving times. A near syncopal event while driving may result in a motor vehicle accident, but we did not collect data on near syncope. We do not have data on the rate of post mortem interrogations. Earlier ICDs were not equipped with EGM storage, and the programming options were limited. We collected data back to 1997 and ascertained that all ICDs in this study were equipped with EGMs. However, a more contemporary cohort would provide a more accurate estimation of the current risks. It is important to compare the rate of syncope between inappropriate and appropriate therapies, and the risk of harm. In the present study, however, we focused on the rate of syncope associated with inappropriate ICD therapy. Actually, we examined the number of appropriate therapies in this population, but we did not try to determine the rate or cause of syncope associated with appropriate ICD therapies. We will examine the rate of syncopal events associated with appropriate ICD therapy in the near future.

## Conclusions

We demonstrated that a small number of patients (0.7%) experienced syncope associated with inappropriate ICD therapies, with an estimated maximum annual risk of harm that was far below the commonly cited acceptable risk threshold of 5 in 100,000. This observation suggests that in the case of private driving habits, there may be no need for driving restrictions following inappropriate ICD therapies, if it is not associated with a loss of consciousness.

## Electronic supplementary material

ESM 1 (DOCX 44 kb)
