# Supplementary material for: Inappropriate implantable cardioverter defibrillator shocks—incidence, effect, and implications for driver licensing
Source: J Interv Card Electrophysiol. 2017 Jul 20;49(3):271–80. doi: 10.1007/s10840-017-0272-4 (PMC5543197; doi:10.1007/s10840-017-0272-4)
Supplement: Supplementary file 1 — (DOCX 44 kb) [file 10840_2017_272_MOESM1_ESM.docx]

Inappropriate implantable cardioverter-defibrillator shocks - incidence, effect and implications for driver licensing (JICE-D-17-00129 R2)

Eiichi Watanabe MD, PhD

**Supplementary File**

**1. Participants list**

Masayuki Sakurai, Shinya Shimoshige, Hisashi Yokoshiki, Shingo Sasaki, Koji Fukuda, Hiroshi Yamazaki, Yasushi Imai, Tomoyuki Kabutoya, Shigeto Naito, Kazuo Matsumoto, Yuji Nakasato, Marehiko Ueda, Jun Umemura, Yoshiyasu Aizawa, Harumizu Sakurada, Seiji Fukamizu, Yoichi Kobayashi, Iku Tannno, Teiichi Yamane, Shinichi Niwano, Kenji Kurosaki, Toshiyuki Ishikawa, Hisa Shimojima, Mitsuhiro Nishizaki, Hiroshi Furushima, Masahito Sato, Takahiro Takeuchi, Yasuya Inden, Yukihiko Yoshida, Satoshi Shizuta, Takeshi Shiroyama, Koichi Inoue, Yoshio Furukawa, Akihiro Yoshida, Nobuhiro Nishii, Katsuhiko Imai, Akihiko Shimizu, Takayuki Nagai, Kenji Ando, Masahiro Ogawa, Toshihiro Honda, Hisao Ogawa, Kazuhito Hirata, Keiji Inoue, Mitsuyuki Ito, Shinji Ichikawa, Koji Nitta, Kunihiko Tsuchiya, Taizo Okuda, Hideo Nishikawa, Masato Tsujii, Ryosuke Kametani, Susumu Iwafuji, Yoshihisa Abe, Ken Terata, Koichiro Yoshioka, Kazutaka Aonuma, Tetsuya Haruna, Kazuyuki Tanoue, Masuyuki Sato, Yoshihiro Sobue, Masaru Yamamoto, Asami Fujiwara, and Riiko Asanuma.

**2. Figure. An annual number of implantations of the ICDs (n=417)**

The median date of the implantation was November 2010. It showed that 88% of patients received ICDs after 2005.
